# Cost-utility analysis of three PD-1 inhibitors combined with chemotherapy in the first-line treatment of locally advanced or metastatic esophageal squamous carcinoma in China

**DOI:** 10.1186/s13561-026-00784-2

**Published:** 2026-05-09

**Authors:** Li Liao, Jing Gong

**Affiliations:** Department of Pharmacy, Changshou District Hospital of Traditional Chinese Medicine, Chongqing, China

**Keywords:** Sintilimab, Camrelizumab, Toripalimab, Chemotherapy, Esophageal squamous carcinoma, Cost-utility analysis

## Abstract

**Objective:**

To evaluate the economics of sintilimab combination chemotherapy versus chemotherapy and sintilimab combination chemotherapy versus camrelizumab/ toripalimab combination chemotherapy in the first-line treatment of locally advanced or metastatic esophageal squamous carcinoma (ESCC) from the perspective of the Chinese health system.

**Methods:**

A partitioned survival model (PSM) with a tracked time horizon of 5 years was developed. In the basal analysis, a direct cost-utility comparison of sintilimab + chemotherapy vs. chemotherapy in first-line treatment of advanced ESCC was performed based on survival data from the ORIENT-15 trial. Cost and utility value data were obtained from relevant databases or published literature, and the robustness of the model was assessed using one-way sensitivity analysis and probabilistic sensitivity analysis (PSA). In the exploratory analysis, the cost-utility of sintilimab + chemotherapy vs. camrelizumab/ toripalimab + chemotherapy was indirectly compared by Network Meta-Analysis (NMA) combined with hazard ratio (HR).

**Results:**

Sintilimab in combination with chemotherapy resulted in 0.33 quality-adjusted life years (QALYs) more than chemotherapy with an incremental cost-effectiveness ratio (ICER) of 25,409.27 USD/QALY, which was lower than the willingness-to-pay (WTP). Sensitivity analysis showed that the utility value of stable disease and the price of sintilimab had the greatest impact on outcome stability. Exploratory analysis indicate that toripalimab plus chemotherapy yields an additional 0.04 QALYs compared with sintilimab plus chemotherapy, with an ICER of 9,953.24 USD/QALY. In contrast, camrelizumab plus chemotherapy yields 0.05 fewer QALYs compared with sintilimab plus chemotherapy.

**Conclusion:**

For the first-line treatment of locally advanced or metastatic ESCC, the addition of sintilimab to conventional chemotherapy regimens was economical. Among similar PD-1 inhibitors, the toripalimab plus chemotherapy regimen is more costly but more effective than the sintilimab plus chemotherapy regimen, and is cost-effective at the WTP threshold of 3 × GDP per capita. In contrast, the camrelizumab plus chemotherapy regimen is dominated (higher cost and worse effectiveness) by the sintilimab plus chemotherapy regimen.

 According to the global cancer data in 2020, esophageal cancer (EC), which is one of the most common malignant tumors in the world, ranks 7th (3.1%) and 6th (5.5%) in incidence and mortality of all cancer types respectively, with about 604,000 new cases and 544,000 deaths worldwide [1].

EC comprises two histological types, ESCC and esophageal adenocarcinoma (EAC), which have distinct biological characteristics, geographic distributions, and risk factors. In some developing countries such as Asia, ESCC is the predominant histological subtype. China is one of the regions with the highest risk of developing EC, accounting for about half of the global incidence, of which more than 90% is ESCC, with tobacco and alcohol abuse being the most common risk factors [2, 3]. Lack of awareness of early screening and insidious symptoms of EC, most patients are diagnosed at an advanced stage, resulting in a lower incidence of EC in China than in the UK and the US, but a higher mortality rate than in the UK and the US, which is an important cause of cancer-related deaths in China [4].

ESCC has a high degree of malignancy and a high rate of recurrence after treatment, which imposes a heavy disease and economic burden on society. A multicenter retrospective study of EC patients diagnosed in China from 2002 to 2011 showed that the overall average medical expenditure for EC patients in China was USD 38,666, and this value has increased significantly, with drug expenditure accounting for about 45% of total medical expenditure in China [5]. Results of another dynamic cohort modeling study on the disease and economic burden of EC in China suggested that between 2013 and 2030, the incidence of EC will increase from 61 to 64.5 cases per 100,000, with 11.5% new cases per year, and direct medical expenditures will increase by 128.7% (from $3.34 billion to $76.4 billion), an annual growth ratio of 5% [6].

Before the advent of immunotherapeutic agents, systemic treatment of advanced ESCC was dominated by cytotoxic agents. Paclitaxel or fluorouracil combined with platinum was always regarded as the standard of care. The long-term survival benefit for patients with advanced ESCC receiving chemotherapy is not satisfactory and the prognosis is poor, with an average survival time of only 7–13 months and a 5-year survival rate of < 5% [7, 8].

Immune checkpoint inhibitors (ICIs) that block the PD-1/programmed cell death ligand 1 (PD-L1) pathway have revolutionized the treatment outlook for many cancers in recent years by providing increased survival benefits to patients. Approximately half of ESCC patients have PD-L1/PD-L2 expression, indicating that ESCC patients are potential beneficiaries of PD-1/PD-L1 inhibitors [9]. Several random clinical trials have also demonstrated the efficacy of PD-1/PD-L1 inhibitors in the treatment of advanced or metastatic ESCC, such as nivolumab, pembrolizumab, and camrelizumab. The treatment of advanced ESCC has fully entered the era of “immunotherapy” [10–13].

Sintilimab [14], camrelizumab [15] and toripalimab [16] are fully humanized IgG4 anti-PD-1 monoclonal antibodies developed in China with a similar mechanism of action: binding to PD-1 (expressed on activated T lymphocytes, B cells, and natural killer cells), thereby blocking the interaction of PD-1 with its ligand PD-L1 (overexpressed on some cancer cells) and PD-L2 (mainly expressed on antigen-presenting cells), reactivating the anti-tumor activity of lymphocytes.

ORIENT-15 [17]、ESCORT-1st [10], and JUPITER-06 [13] were multicenter, double-blind phase III randomized controlled clinical trials that evaluated the efficacy and safety of sintilimab, camrelizumab, and toripalimab in combination with chemotherapy (paclitaxel and cisplatin) in the first-line treatment of Chinese patients with advanced ESCC, respectively. Compared with chemotherapy, the combination of these three PD-1 inhibitors prolonged patients’ median progression-free survival (PFS) by 0.2–1.5 months, median overall survival (OS) by 3.3-6.0 months, reduced the risk of death by 30%-42% and the rate of serious adverse events (SAEs) by 3.2%-5.0%. Based on the results of these three clinical trials, sintilimab, camrelizumab, and toripalimab in combination with chemotherapy were approved for the first-line treatment of unresectable locally advanced or metastatic ESCC in China.

The Medicare prices of these three PD-1 inhibitors are much lower than their imported counterparts (pembrolizumab, nivolumab), but the economics of the three immunotherapy regimens in advanced ESCC remains to be evaluated. Therefore, the primary aim of this study was to directly compare the economics of sintilimab combined with chemotherapy (STP) compared with paclitaxel/5-fluorouracil (5-FU) plus cisplatin (TP) in the first-line treatment of advanced ESCC, and the secondary aim was to indirectly compare the economics of sintilimab with the similar drugs camrelizumab (CTP) and toripalimab (TTP) combined with chemotherapy, to provide a reference for health insurance decisions and rational clinical use of drugs.

## Materials and methods

### Model structure

**Fig. 1:**
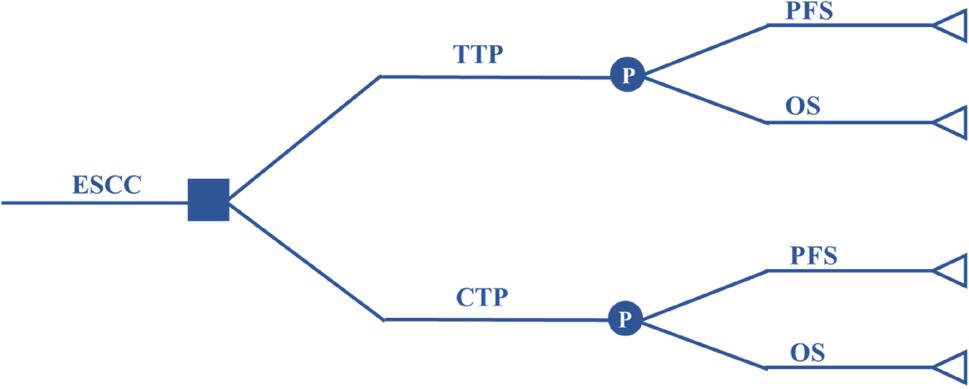
A partitioned survival analysis (PartSA) model simulating esophageal squamous cell carcinoma (ESCC). STP. sintilimab plus chemotherapy: TP, paclitaxel/5-fluorouracil plus cisplatin; TTP, toripalimab plus chemotherapy: CTP, camrelizumab plus chemotherapy: PFS, progression-free survival: PD, progressive disease: OS, overall survival.

**Fig. 2:**
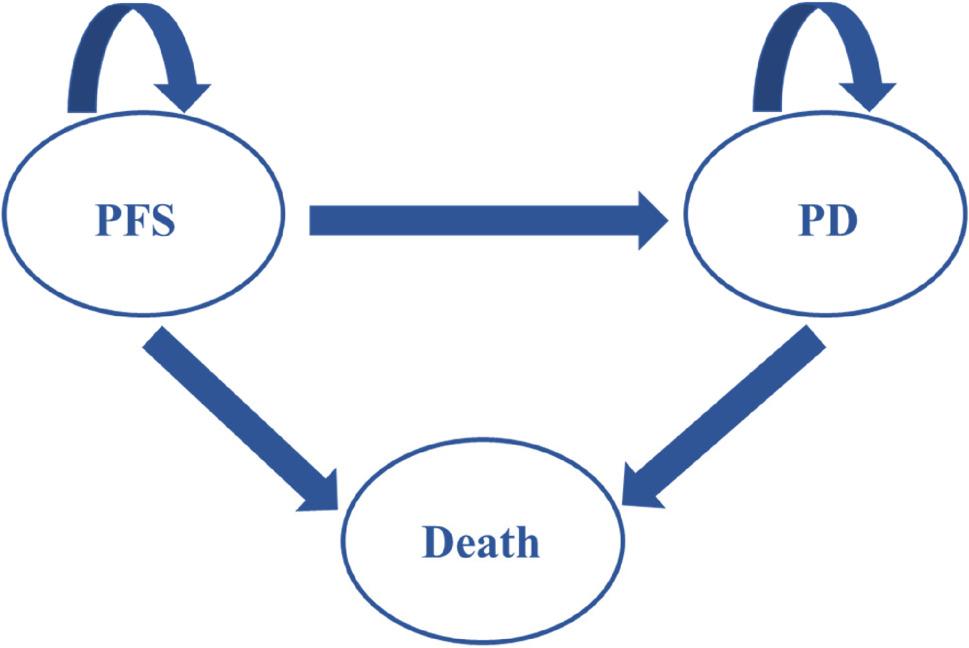
A three-state transition model. PFS, progression-free survival: PD, progressive disease

A PSM (Fig. 1) was developed using TreeAge Pro 2020, which contained three mutually exclusive health states: progression-free survival (PFS), progressive disease (PD), and death. The initial health state for all patients was PFS, and patients remained in this state or progressed to the next health state during each cycle, with the final health state being death (Fig. 2).

Considering the short survival of advanced ESCC, the tracked time horizon of the model was 5 years. The PSM has a cycle of 21 days in line with the dosing cycle. Model outcomes were expressed as cumulative costs, life years (LYs), QALYs, and ICER. Costs and utilities were discounted at 5% according to *the China Guidelines for Pharmacoeconomic Evaluations* (2020), and the WTP threshold was 3 times the gross domestic product (GDP) per capita in China in 2021 (35,427.74 USD).

### Intention-to-treat population

The population included in this study was consistent with the clinical trials ORIENT-15, JUPITER-06, and ESCORT-1st. Patients > 18 years of age with a histologically or cytologically diagnosed unresectable locally advanced, recurrent, or metastatic ESCC, who have not received systemic therapy, have at least one measurable lesion, and an Eastern Cooperative Oncology Group (ECOG) performance status (PS) score of 0–1.

### Clinical data

**Fig. 3:**
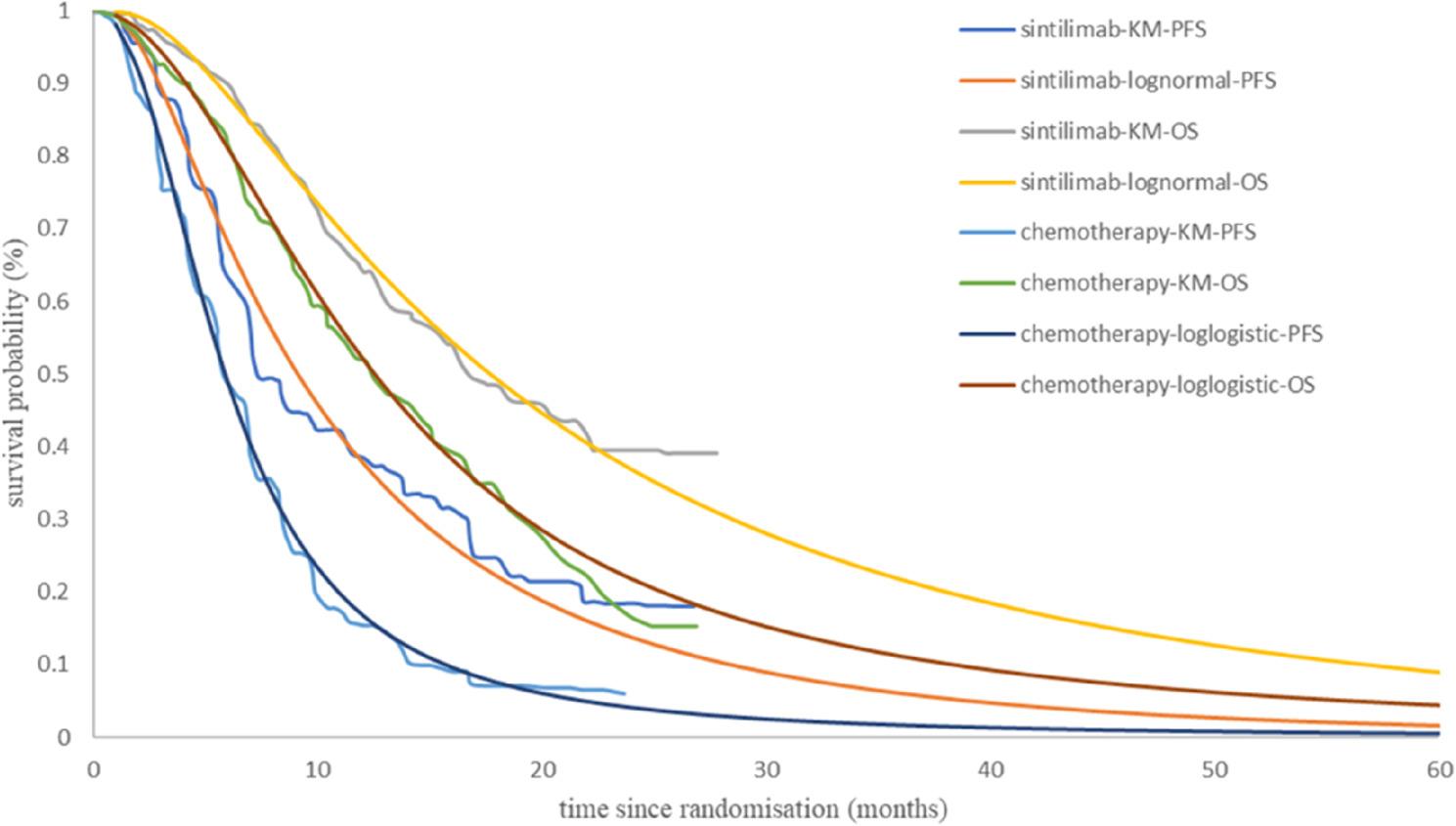
Kaplan-Meier Curve Fitting and Extrapolation in all patients. PFS, progression-free survival; OS, overall survival

In this study, data points were extracted from the ORIENT-15 trial using the Engauge Digitizer (http://digitizer.sourceforge.net) software, and the survHE package in R software (version 4.2.1; https://www.r-project.org) was used for reconstruction of individual survival data, due to the limited follow-up time of the clinical trial and the unavailability of individual patient survival data. The reconstructed data were fitted and extrapolated using exponential, GenGamma, Gompertz, Weibull, log-logistic, and lognormal functions. The survival function with the best fit was selected according to the Akaike information criterion (AIC) and the Bayesian information criterion (BIC) combined with the visual test. The best fit of the PFS and OS curves for the STP group was the lognormal function [S(t) = 1-φ {(Int-µ)/σ}], and the PFS and OS curves in the TP group were best fit as a log-logistic function [S(t) = 1/(1 + λtγ)] (Fig. 3).

### Medical cost

Only direct medical costs were calculated in this study, including the cost of drugs, administration of SAEs, the laboratory and imaging tests during follow-up, consultation, bed, intravenous configuration and infusion during hospitalization, and treatment after disease progression. The costs in the model were converted to US dollars (1 USD = 6.857 CNY).

Prices for sintilimab, camrelizumab, and toripalimab were used as published by Medicare in 2021. The prices of paclitaxel and cisplatin used the average of the drug winning bid prices by province in the MENET (https://www.menet.com.cn). Dosing and regimens were consistent with clinical trials ORIENT-15, ESCORT-1st, and JUPITER-06. Sintilimab (200 mg), camrelizumab (200 mg), toripalimab (240 mg), or placebo in combination with chemotherapy [cisplatin (75 mg/m^2^) and paclitaxel (175 mg/m^2^)] was administered every 3 weeks for 6 cycles of treatment followed by maintenance treatment with sintilimab or camrelizumab or toripalimab or placebo until the development of unacceptable toxicity, the disease progression, investigator decision, or death, etc. Maintenance therapy cannot exceed 2 years. To calculate the dose of chemotherapeutic agents used, the mean body surface area (BSA) of patients in this study was used as the Chinese per capita BSA (1.72 m^2^).

Grade 1–2 adverse events usually do not require management. The incidence of SAEs, except for anemia, lymphopenia, and neutropenia, was low and the cost of treatment was negligible. Therefore, only the treatment costs for grade 3 or higher anemia, lymphopenia, and neutropenia were calculated in this model. The incidence of SAEs in the four groups was obtained from the ORIENT-15, ESCORT-1st, and JUPITER-06 trials. Treatment costs were taken from literature data. Costs of SAEs were calculated only in the first cycle of the model run.

Laboratory tests during routine follow-up included routine blood, urine, stool, biochemical complete set, thyroid function test, coagulation function test, electrocardiogram and oncology-related index test, and regular imaging (CT/PET-CT) for efficacy assessment during treatment.

Patients continued with other antitumor therapies (chemo-, targeted-, or immunotherapy) or the best supportive care (BSC) after progression on the first-line treatment. ORIENT-15, ESCORT-1st and JUPITER-06 trials did not provide treatment modalities for patients after progression to first-line therapy. Therefore, the cost of first-line treatment after progression in this study was taken from the relevant literature.

The cost parameters and the incidence of SAEs for each regimen were shown in Table 1.

**Table 1:** Model inputs

Variable	Baseline value	Range	Distribution	Source
Kaplan-Meier survival curves Fitting Parameters				
Log-normal PFS survival model with STP	µ = 2.2096; σ = 0.8904; AIC = 1308.180; BIC = 1315.760;	-	-	-
Weibull PFS survival model with STP	γ = 1.3912; λ = 0.0286; AIC = 1338.955; BIC = 1346.535;			-
Log-logistic PFS survival model with TP	γ = 2.2348; λ = 0.1702; AIC = 1396.980; BIC = 1404.590;	-	-	-
Log-normal OS survival model with STP	µ = 2.8726; σ = 0.9101; AIC = 1208.160; BIC = 1215.740;	-	-	-
Weibull OS survival model with STP	γ = 1.6146; λ = 0.0068; AIC = 1217.126; BIC = 1224.706;			-
Log-logistic OS survival model with TP	γ = 1.9688; λ = 0.0799; AIC = 1498.955; BIC = 1506.565;	-	-	-
OS HR network meta-analysis of TTP versus STP	0.92	(0.33,2.50)	-	NMA
OS HR network meta-analysis of CTP versus STP	1.1	(0.40,3.10)	-	NMA
PFS HR network meta-analysis of TTP versus STP	1.0	(0.36,3.00)	-	NMA
PFS HR network meta-analysis of CTP versus STP	1.0	(0.34,2.90)	-	NMA
Costs (USD)				
Sintilimab (10 ml/100 mg)	157.50	126.00-189.00	gamma	MENET
Camrelizumab (200 mg)	427.01	341.61-512.41	gamma	MENET
Toripalimab (6 ml/240 mg)	278.98	223.18-334.78	gamma	MENET
Paclitaxel (5 ml/30 mg)	49.71	39.77–59.66	gamma	MENET
Cisplatin (10 ml/10 mg)	4.36	3.49–5.24	gamma	MENET
5-fluorouracil (250 mg)	6.80	5.44–8.16	gamma	MENET
Subsequent therapy cost per cycle	150.14	120.11-180.17	gamma	(23)
Routine follow-up cost per cycle	357.34	285.87-428.81	gamma	(20)
Hospital management cost per cycle	69.81	55.85–83.77	gamma	(26)
Treatment cost of anemia	508.20	381.20-653.30	gamma	(21)
Treatment cost of decreased WBC count	466.00	372.80-559.20	gamma	(21)
Treatment cost of neutropenia	466.00	372.80-559.20	gamma	(21)
Utility				
PFS	0.741	0.593–0.889	beta	(18)
PD	0.581	0.465–0.697	beta	(18)
Disutility				
Anemia	-0.074	-0.110 to -0.037	beta	(29)
Decreased WBC count	-0.09	-0.12 to -0.059	beta	(25)
Neutropenia	-0.09	-0.12 to -0.059	beta	(29)
Incidence of SAEs (grade ≥ 3)				
Anemia of STP group	0.13	0.104–0.156	beta	(17)
Decreased WBC count of STP group	0.17	0.136–0.204	beta	(17)
Neutropenia of STP group	0.30	0.240–0.360	beta	(17)
Anemia of CTP group	0.17	0.136–0.204	beta	(10)
Decreased WBC count of CTP group	0.24	0.192–0.288	beta	(10)
Neutropenia of CTP group	0.40	0.320–0.480	beta	(10)
Anemia of TTP group	0.11	0.088–0.132	beta	(13)
Decreased WBC count of TTP group	0.20	0.160–0.240	beta	(13)
Neutropenia of TTP group	0.42	0.336–0.504	beta	(13)
Anemia of TP group	0.10	0.080–0.120	beta	(17)
Decreased WBC count of TP group	0.22	0.176–0.264	beta	(17)
Neutropenia of TP group	0.34	0.272–0.408	beta	(17)
Other				
Discount	5%	0–8%	-	-

*STP* sintilimab plus chemotherapy, *TP* paclitaxel/5-fluorouracil plus cisplatin, *TTP* toripalimab plus chemotherapy, *CTP* camrelizumab plus chemotherapy, *AIC* Akaike information criterion, *BIC* Bayesian information criterion, *PFS* progression-free survival, *PD* progressive disease, *WBC* white blood cell, *SAEs* serious adverse events

### Health utility

Utility values of patients were not reported in the ORIENT-15, ESCORT-1st, and JUPITER-06 clinical trials. Referring to other pharmacoeconomic evaluation studies related to ESCC, the utility values in this model were taken from a global multicenter study of utility values in patients with gastric cancer and adenocarcinoma of the gastroesophageal junction, with a utility value of 0.741 for PFS status and 0.581 for PD status [18].

The utility value data were presented in Table 1.

### Sensitivity analysis

To test the robustness of the model, we conducted a one-way sensitivity analysis and PSA.

The ranges of the parameters were set to ± 20% of the base values, except for the utility values of SAEs and the discount rate (0–8%) in the one-way sensitivity analysis. The result of the one-way sensitivity analysis was presented as a tornado plot.

The distribution types and value ranges of the relevant parameters were shown in Table 1.

PSA was used to assess the effect of overall changes in the model parameters on the results. We set the cost parameters as gamma distribution. The health utilities and the incidence of SAEs were set as beta distribution. Monte Carlo simulations were run 1,000 times. PSA results were presented as the incremental cost-effectiveness scatter plot and cost-effectiveness acceptability curve (CEAC).

### Subgroup analysis

**Fig. 4:**
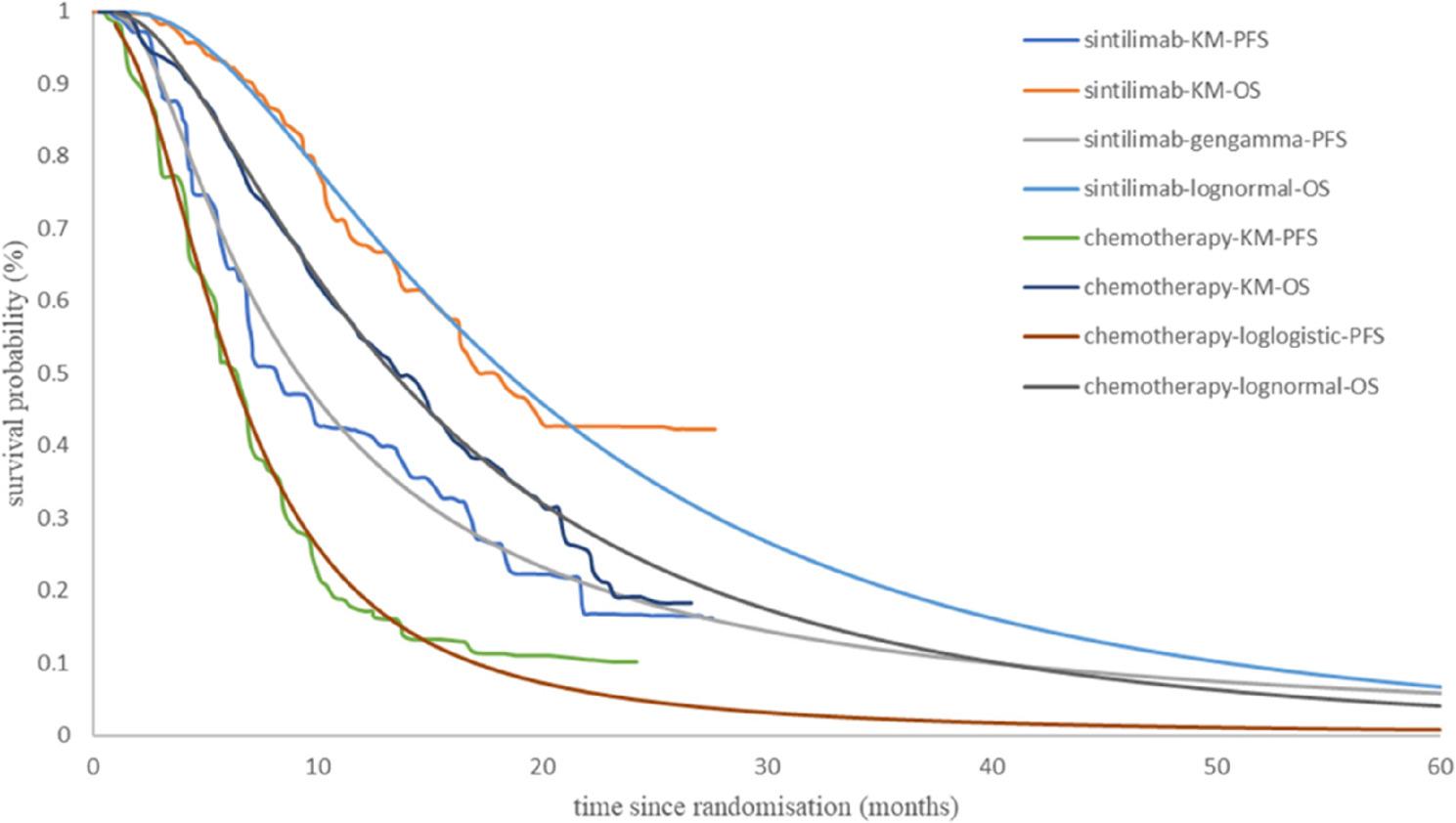
Kaplan-Meier Curve Fitting and Extrapolation in patients with combined positive scores of ≥10 for expression of programmed cell death ligand 1. PES, progression-free survival: OS, overall survival

PD-L1 expression in tumor cells may affect the efficacy of PD-1 inhibitor therapy. Survival data were extracted from the PFS and OS curves of the ORIENT-15 with combined positive fraction (CPS) ≥ 10, and curve fitting and extrapolation were performed. In a subgroup analysis, the cost-utility of STP vs. TP in ESCC patients with PD-L1 tumor CPS ≥ 10 was assessed. In patients with CPS ≥ 10, the PFS curve was the best fit as a GenGamma function in the STP group, the PFS curve was the best fit as a log-logistic function in the TP group, and the OS curves were the best fit as a lognormal function in the STP and TP groups (Fig. 4).

### Exploratory analysis

The ORIENT-15, ESCORT-1st, and JUPITER-06 trials controls were all placebo combined with chemotherapy and lacked a “head-to-head” clinical trial of STP, CTP, and TTP in the first-line treatment of advanced ESCC. To directly compare the cost-effectiveness of these three treatment options, we adopted the methodology used by Hoyle et al. [19] in their pharmacoeconomic evaluation of advanced renal cell carcinoma, employing a NMA to construct a comparative network. We designated STP as the control group and calculated the HR for PFS and OS of CTP and TTP relative to STP. Additionally, for computational feasibility, we fitted the PFS and OS curves of the STP group to a Weibull distribution, obtaining two parameters of this distribution: the scale parameter (λ) and the shape parameter (γ). Subsequently, we used the following formula to derive the model parameters for the CTP and TTP groups:

$$\gamma_{trial\;group}=\gamma_{control\;group}$$

$$\lambda_{trial\;group}=\lambda_{control\;group}*HR$$

The curve fitting parameters and the combined HR results were shown in Table 1.

## Results

### Basic analysis

The STP group could obtain 1.98 LYs and 1.33 QALYs, corresponding to a total cost of 25,437.35 USD. The TP group could obtain 1.52 LYs and 0.99 QALYs, corresponding to a total cost of 17,006.05 USD. Compared with the TP group, the STP group had an incremental cost of 8,431.29 USD and an incremental QALY gain of 0.33 QALYs, resulting in an ICER of 25,409.27 USD/QALY. The ICER was below the WTP, indicating that STP was cost-effective (Table 2).

**Table 2:** Results

	Cost ($)	LYs	QALYs	ICER
Basic Analysis				
STP	25437.35	1.98	1.33	25409.27
TP	17006.05	1.52	0.99	-
Subgroup Analysis				
STP	24235.41	1.97	1.35	21782.96
TP	17617.29	1.60	1.05	-
Exploratory Analysis				
STP	23764.63	1.74	1.17	-
TTP	24195.06	1.80	1.21	9953.24
CTP	25137.71	1.63	1.11	dominated

*STP* sintilimab plus chemotherapy, *TP* paclitaxel/5-fluorouracil plus cisplatin, *TTP* toripalimab plus chemotherapy, *CTP* camrelizumab plus chemotherapy, *LYs* life years, *QALYs* quality-adjusted life-years, *ICER* incremental cost-effectiveness ratio

### One-way sensitivity analysis

**Fig. 5:**
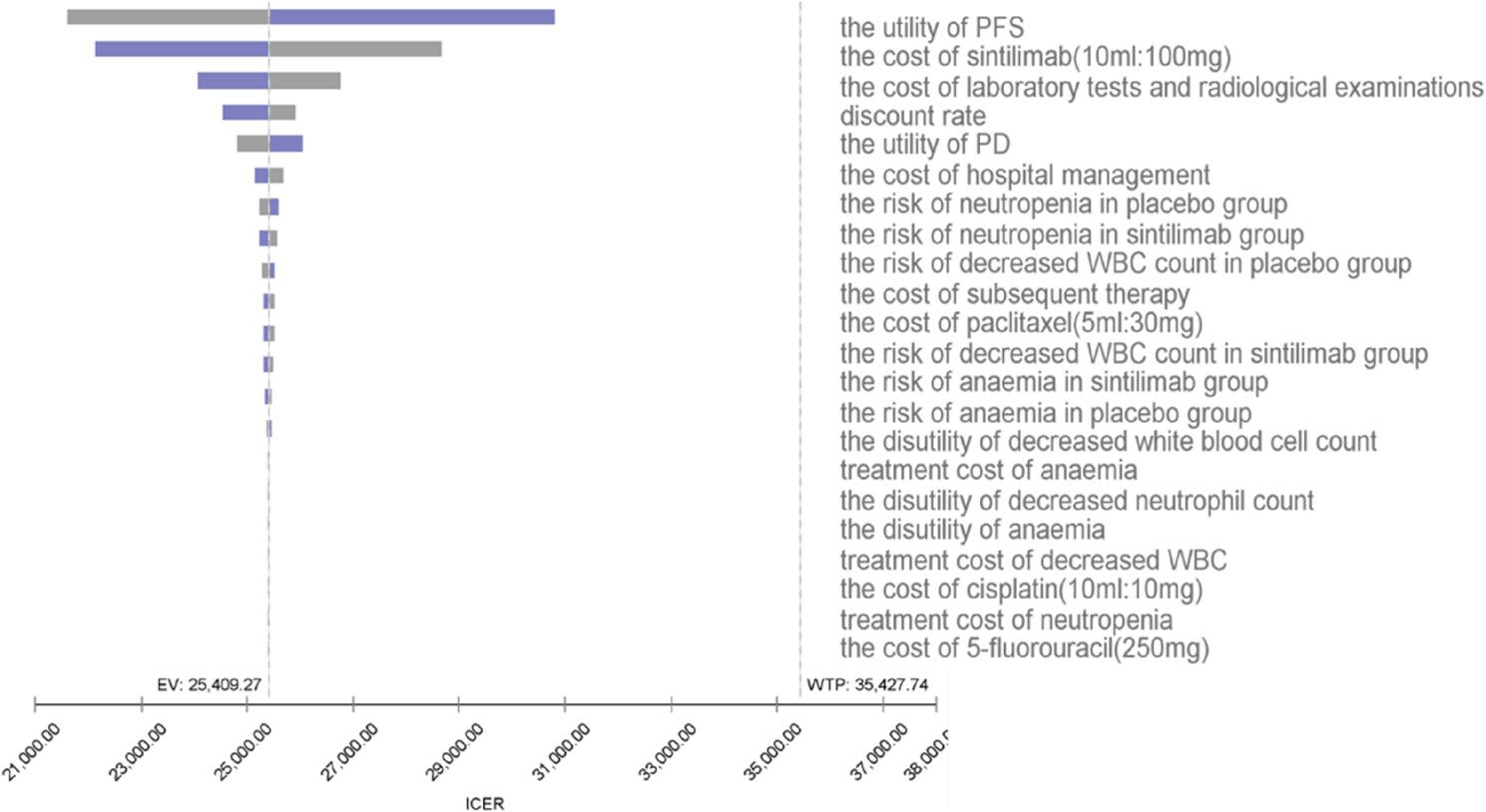
One-way sensitivity analysis for sintifimab + chemotherapy compared with placebo + chemotherapy. PES, progression free survival; PD, progressive disease; WBC, white blood cell; ICER, incremental cost effectiveness ratio; EV, expected value; WTP, willingness-to-pay.

From the tornado plot (Fig. 5), it can be seen that the model parameters that had the greatest impact on the results were the utility value of PFS and the price of sintilimab. The cost of routine tests performed during follow-up, the discount rate and the utility value of PD also had a greater impact on the model. The cost during hospitalization, the incidence of SAEs, subsequent therapy after disease progression, and the cost of SAEs had essentially no effect on the results. Regardless of how the input model parameters vary within their ranges, the ICER values were lower than the WTP and the final conclusions do not change.

### Probabilistic sensitivity analysis

**Fig. 6:**
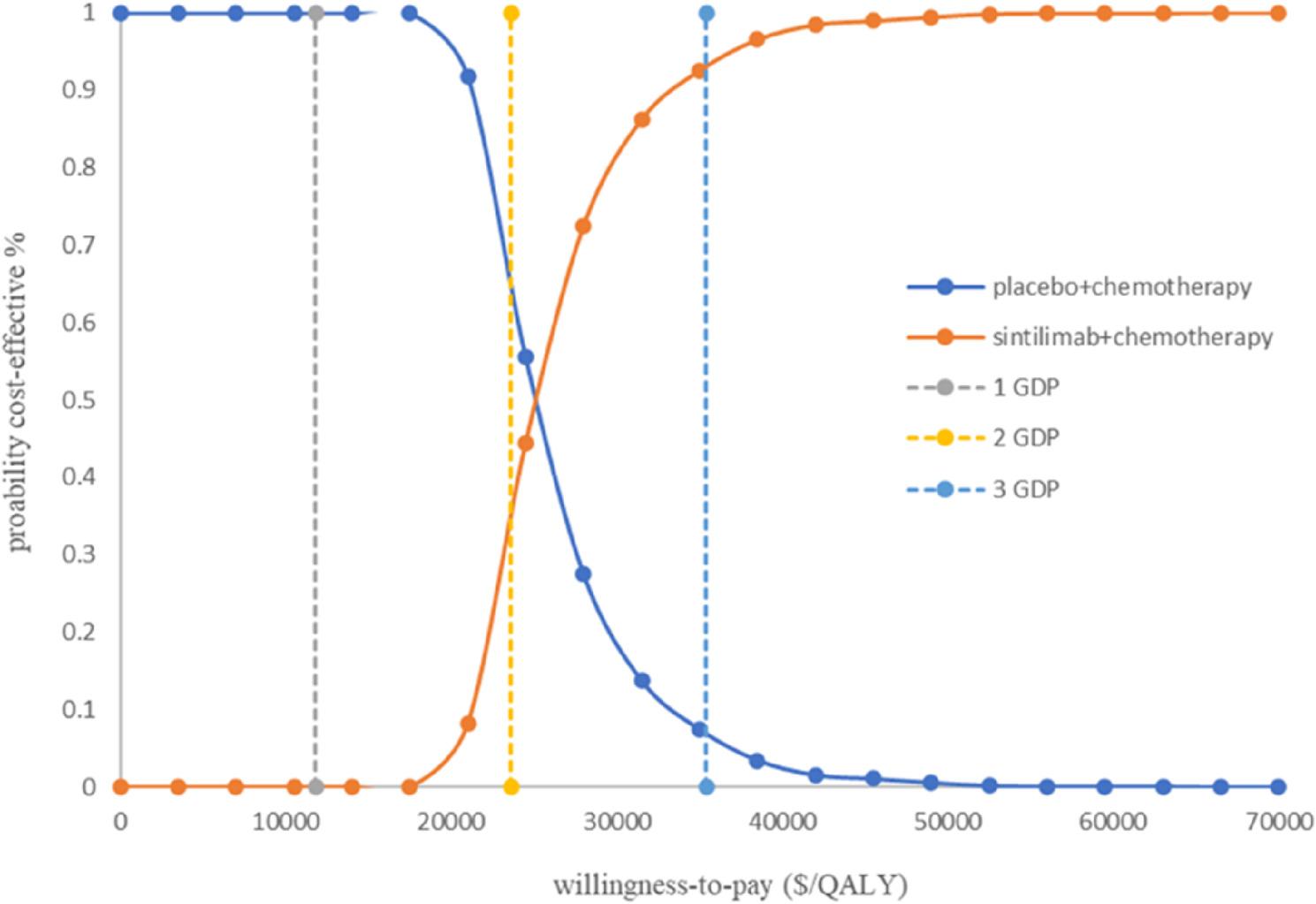
Cost-effectiveness acceptability curves (CEAC) for sintilimab + chemotherapy compared with placebo + chemotherapy.

**Fig. 7:**
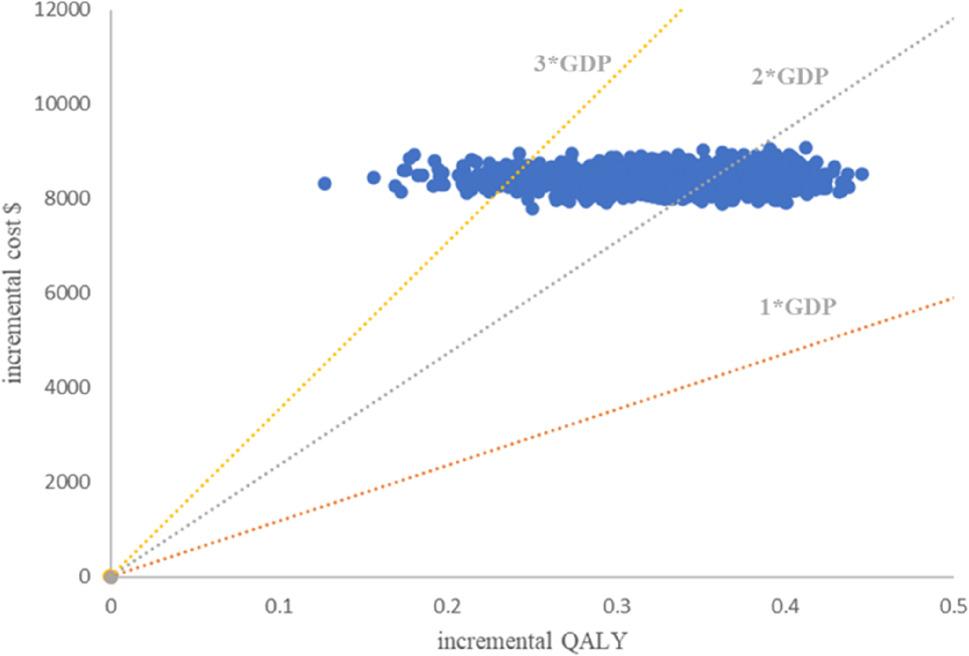
Probability sensitivity analysis scatter plot for sintilimab chemotherapy compared with placebo + chemotherapy

From the CEAC (Fig. 6), it can be seen that the probability of the STP being economic at a WTP setting of 3 times GDP per capita was 75%, and the probability of the STP scenario being economic at a WTP of 66,500 USD was 100%, which was consistent with the results of the incremental cost-effectiveness scatter plot (Fig. 7).

### Subgroup analysis

In patients with PD-L1 CPS ≥ 10, the STP group could obtain 1.97 LYs and 1.60 QALYs, corresponding to a total cost of 24,235.41 USD. The TP group can obtain 1.60 LYs and 1.05 QALYs, corresponding to a total cost of 17,617.29 USD. The incremental cost of STP vs. TP was 6,618.12 USD, incremental QALYs was 0.30 QALYs, and ICER was 21,782.96 USD/QALY, which was lower than the WTP (Table 2).

### Exploratory analysis

In the intention-to-treat population, the STP group obtained 1.74 LYs and 1.17 QALYs, corresponding to a total cost of 3,764.63 USD. The TTP group yielded 1.80 LYs and 1.21 QALYs, corresponding to a total cost of 24,195.06 USD. The CTP group received 1.63 LYs and 1.11 QALYs, corresponding to a total cost of 25,137.71 USD. The incremental cost and incremental QALYs of TTP vs. STP were 430.43 USD and 0.04 QALYs, respectively, and the ICER is 9,953.24 USD/QALY, which was lower than the WTP. Therefore, the TTP plus chemotherapy regimen is cost-effective. The incremental cost and incremental QALYs for CTP vs. STP were 1,373.08 USD and − 0.05 QALYs, indicating higher costs and poorer outcomes; therefore, the CTP plus chemotherapy regimen is dominated by STP. In this case, there is no need to calculate the ICER (as a negative value is not meaningful) (Table 2).

## Discussion

ESCC is insidiously symptomatic, and most patients are advanced at the time of diagnosis. In clinical practice, many patients have a poor general condition, inadequate organ function, or other comorbidities that make them unable to tolerate the standard chemotherapy regimen of paclitaxel/5-FU in combination with platinum, as well as a high postoperative recurrence rate. All of these factors contribute to the poor prognosis of advanced ESCC. In recent years, the rapid development of ICIs, such as PD-1/PD-L1 and cytotoxic T-lymphocyte antigen 4 (CTLA-4), has brought more survival benefits to ESCC patients, extending OS while improving quality of life.

Both the imported nivolumab and pembrolizumab, as well as the domestic sintilimab, toripalimab and camrelizumab, have shown significantly better PFS and OS benefits than conventional chemotherapy in the treatment of ESCC patients, but the better efficacy corresponds to a heavy economic burden. Therefore, when choosing PD-1 inhibitors, efficacy and affordability are equally important.

Liu S et al. [20–22] pharmacoeconomic studies on nivolumab, and Zhan M et al. [23–25] on pembrolizumab in the first- and second-line treatment of advanced ESCC showed that the ICER values of these two imported PD-1 inhibitors in combination with chemotherapy compared with chemotherapy regimens were 4–10 times higher than the current WTP in China, and the probability of having economic viability in China was 0, limiting the use of immunotherapeutic agents in Chinese ESCC patients. The number of lines of treatment differs between camrelizumab and toripalimab combined with chemotherapy in the treatment of ESCC, as well as their economics. Toripalimab combined with chemotherapy for the first-line treatment of ESCC was economical [26]. First-line treatment of ESCC with camrelizumab in combination with chemotherapy was not economically feasible [27]. There was an economic regimen for the second-line treatment of ESCC with camrelizumab alone [28–30]. There are no pharmacoeconomic studies on sintilimab alone or in combination with chemotherapy for ESCC.

In this study, a PSM was used to assess the economics of sintilimab in combination with chemotherapy in the first-line treatment of advanced, recurrent, or metastatic ESCC from a Chinese health system perspective. The results of the basic analysis showed that, when the WTP threshold was set at 3 times the 2021 GDP per capita, adding sintilimab to the first-line treatment regimen for advanced ESCC yielded 0.33 more QALYs and increased medical costs, but the ICER remained below the WTP threshold. Therefore, the regimen was economic and sintilimab in combination with chemotherapy could be a new first-line treatment option for patients with advanced ESCC in China.

In the one-way sensitivity analysis, the tornado plot showed that the utility value of PFS had the greatest effect on the outcome, and that health utility values differed substantially across countries. Currently, only one of the published pharmacoeconomic studies [28] on PD-1 inhibitors for ESCC has a utility value using data published in phase III clinical trials of the drug. And only the utility values at baseline for patients, assuming a linearly proportional decrease in utility values from disease progression to death. The health utility values for PFS and PD status in other pharmacoeconomic studies of PD-1 inhibitors were derived from a global multicenter quality-of-life study of a population with adenocarcinoma of the stomach or gastroesophageal junction. The Chinese population was not included in this study, and the results may not match the actual health utility values of Chinese ESCC patients. This may have implications for assessing the cost-utility of STP in Chinese ESCC patients. In addition to the utility value of PFS, the price of sintilimab had a large impact on the model. In contrast, in the pharmacoeconomic evaluation of imported PD-1 inhibitors, it is usually the price of the immune drug that has the greatest impact on the model results. This may be due to the narrowing of the gap between the price of sintilimab and the other medical costs spent throughout the disease process after its price reduction. The survival benefits of imported PD-1 inhibitors do not compensate for their economics disadvantage. The launch of domestic PD-1 inhibitors has undoubtedly brought a new option for ESCC patients, with better efficacy than conventional chemotherapy, no new safety events, and lower ICERs than the current WTP in China. The final results of the model did not change regardless of the fluctuation of the model parameters within its range, indicating the robustness of the model.

This study evaluated the cost-utility of STP vs. TP in patients with PD-L1 CPS ≥ 10 in a subgroup analysis. Only 0.03 additional incremental QALYs were available in patients with PD-L1 CPS ≥ 10 compared to the total population, indicating that PD-L1 expression status had no impact on the economics of the STP regimen.

Sintilimab, toripalimab, and camrelizumab have similar mechanisms of action, and the price per cycle is relatively cheap (315 USD, 278.98 USD, and 427.01 USD, respectively). However, there are no “head-to-head” clinical trials of these three drugs in combination with chemotherapy regimens for advanced ESCC, making a direct comparison of their efficacy and affordability difficult. NMA can be based on multiple studies analyzing the results of indirect comparisons between more than two interventions. Therefore, this study used NMA to combine HRs of PFS and OS curves from ORIENT-15, ESCORT-1st and JUPITER-06 in an exploratory analysis to convert γ and λ of the Weibull function in the TTP and CTP groups to obtain extrapolated survival curves to directly compare the cost-utility of the three regimens. The results of the exploratory analysis suggested that, compared with the STP regimen, the TTP regimen appeared to be cost-effective, whereas the CTP regimen was dominated. However, it should be noted that this exploratory analysis relied on indirect comparisons. For survival extrapolation, the STP group was fitted with a Weibull distribution, which may introduce some uncertainty into the final results. Therefore, these finding should be interpreted with caution and require confirmation in future head-to-head trials. After conversion using the Weibull distribution, the ICER values for TTP vs. chemotherapy and CTP vs. chemotherapy were 26,805.9 USD/QALY and 42,706.9 USD/QALY, respectively. These values were generally consistent with the results of two other studies: TTP vs. chemotherapy (ICER = 20,302.54 USD/QALY) [26] and CTP vs. chemotherapy (ICER = 46,671.1 USD/QALY) [27]. The overall findings across studies were not substantially different, suggesting that, compared with chemotherapy, the TTP regimen appeared to be cost-effective, whereas the CTP regimen did not. The use of NMA combined with survival data may provide a useful approach for preliminary economic evaluations in the absence of “head-to-head” clinical trial. However, these results should be interpreted with caution.

The following limitations of this study remain: (1) There are few studies on health-related quality of life in ESCC in China. The health utility values for different disease states in this model were taken from a global multicenter study of utility values for gastric cancer and adenocarcinoma of the gastroesophageal junction. The Chinese population was not included in that study, and the final utility values may not fully reflect the actual situation of ESCC patients in China, which may bias the economic evaluation results (2). Phase III clinical trials have a short follow-up periods, and extrapolation using available survival data could potentially underestimate the efficacy of immunotherapy, which is a recognized limitation of pharmacoeconomic evaluations based on published literature (3). The cost of treatment after the progression of first-line therapy is challenging to estimate accurately. This part of the cost data in this study was taken from the literature, ignoring individual differences, which may introduce bias (4). The exploratory analysis used HR values from NMA combined with survival data, but this approach does not fully account for the heterogeneity among different phase III clinical trials, which may reduce the precision of the results. Despite these limitations, the conclusions drawn in this study can may provide some reference for healthcare decisions and the selection of PD-1 inhibitors for ESCC treatment in the clinical practice.

## Conclusion

In patients with advanced, recurrent, or metastatic ESCC, sintilimab or toripalimab in combination with chemotherapy appeared to provide a survival benefit compared with chemotherapy alone, and was found to be cost-effective at the current WTP threshold (3 × GDP per capita). These regimen may represent new options for the first-line treatment of Chinese patients with advanced ESCC. In contrast, the camrelizumab-containing regimen was dominated (higher cost and lower effectiveness) relative to the sintilimab-containing regimens. Given the exploratory nature of the indirect comparisons used in this study, these findings should be interpreted with caution and require confirmation in future head-to-head trials.

## Data Availability

No datasets were generated or analysed during the current study.

## Abbreviations

ESCC: Esophageal Squamous Carcinoma
PSM: Partitioned Survival Model
PSA: Probabilistic Sensitivity Analysis
NMA: Network Meta-Analysis
HR: Hazard Ratio
QALYs: Quality-Adjusted Life Years
ICER: Incremental Cost-Effectiveness Ratio
WTP: Willingness-To-Pay
PD-1: Programmed Cell Death Protein 1
EC: Esophageal Cancer
EAC: Esophageal Adenocarcinoma
PD-L1: Programmed Cell Death Ligand 1
SAEs: Serious Adverse Events
PFS: Progression-Free Survival
PD: Progressive Disease
LYs: Life Years
GDP: Gross Domestic Product
BSA: Body Surface Area
BSC: Best Supportive Care
CEAC: Cost-Effectiveness Acceptability Curve
CPS: Combined Positive Fraction

## References

[1] Sung H, Ferlay J, Siegel RL, Laversanne M, Soerjomataram I, Jemal A, Bray F. Global Cancer Statistics 2020: GLOBOCAN Estimates of Incidence and Mortality Worldwide for 36 Cancers in 185 Countries. Cancer J Clin. 2021;71(3):209–2492. 10.3322/caac.21660 33538338

[2] Lam AK. Esophageal Squamous Cell Carcinoma. Springer; 2020.

[3] Xie Y, Shi L, He X, Luo Y. Gastrointestinal cancers in China, the USA, and Europe. Gastroenterol Rep (Oxf). 2021;9(2):91–104. 34026216 10.1093/gastro/goab010 PMC8128023

[4] Qiu H, Cao S, Xu R. Cancer incidence, mortality, and burden in China: a time-trend analysis and comparison with the United States and United Kingdom based on the global epidemiological data released in 2020. Cancer Commun (London England). 2021;41(10):1037–48. 10.1002/cac2.12197 PMC8504144 34288593

[5] Guo LW, Huang HY, Shi JF, Lv LH, Bai YN, Mao AY, Liao XZ, Liu GX, Ren JS, Sun XJ, Zhu XY, Zhou JY, Gong JY, Zhou Q, Zhu L, Liu YQ, Song BB, Du LB, Xing XJ, Lou PA, Sun XH, Qi X, Wu SL, Cao R, Lan L, Ren Y, Zhang K, He J, Zhang JG, Dai M, Health Economic Evaluation Working Group CSPiUC. Medical expenditure for esophageal cancer in China: a 10-year multicenter retrospective survey (2002–2011). Chin J Cancer. 2017;36(1):73. 28882179 10.1186/s40880-017-0242-3 PMC5590174

[6] Li Y, Xu J, Gu Y, Sun X, Dong H, Chen C. The Disease and Economic Burdens of Esophageal Cancer in China from 2013 to 2030: Dynamic Cohort Modeling Study. JMIR Public Health Surveill. 2022;8(3):e33191. 34963658 10.2196/33191 PMC8928052

[7] Alsop BR, Sharma P. Esophageal Cancer. Gastroenterol Clin North Am. 2016;45(3):399–412. 27546839 10.1016/j.gtc.2016.04.001

[8] Hirano H, Kato K. Systemic treatment of advanced esophageal squamous cell carcinoma: chemotherapy, molecular-targeting therapy and immunotherapy. Jpn J Clin Oncol. 2019;49(5):412–20. 30920626 10.1093/jjco/hyz034

[9] Baba Y, Nomoto D, Okadome K, Ishimoto T, Iwatsuki M, Miyamoto Y, Yoshida N, Baba H. Tumor immune microenvironment and immune checkpoint inhibitors in esophageal squamous cell carcinoma. Cancer Sci. 2020;111(9):3132–41. 32579769 10.1111/cas.14541 PMC7469863

[10] Luo H, Lu J, Bai Y, Mao T, Wang J, Fan Q, Zhang Y, Zhao K, Chen Z, Gao S, Li J, Fu Z, Gu K, Liu Z, Wu L, Zhang X, Feng J, Niu Z, Ba Y, Zhang H, Liu Y, Zhang L, Min X, Huang J, Cheng Y, Wang D, Shen Y, Yang Q, Zou J, Xu RH. Investigators ES-s. Effect of Camrelizumab vs Placebo Added to Chemotherapy on Survival and Progression-Free Survival in Patients With Advanced or Metastatic Esophageal Squamous Cell Carcinoma: The ESCORT-1st Randomized Clinical Trial. JAMA. 2021;326(10):916–25. 34519801 10.1001/jama.2021.12836 PMC8441593

[11] Kato K, Cho BC, Takahashi M, Okada M, Lin C-Y, Chin K, Kadowaki S, Ahn M-J, Hamamoto Y, Doki Y, Yen C-C, Kubota Y, Kim S-B, Hsu C-H, Holtved E, Xynos I, Kodani M, Kitagawa Y. Nivolumab versus chemotherapy in patients with advanced oesophageal squamous cell carcinoma refractory or intolerant to previous chemotherapy (ATTRACTION-3): a multicentre, randomised, open-label, phase 3 trial. Lancet Oncol. 2019;20(11):1506–17. 31582355 10.1016/S1470-2045(19)30626-6

[12] Sun J-M, Shen L, Shah MA, Enzinger P, Adenis A, Doi T, Kojima T, Metges J-P, Li Z, Kim S-B, Cho BC, Mansoor W, Li S-H, Sunpaweravong P, Maqueda MA, Goekkurt E, Hara H, Antunes L, Fountzilas C, Tsuji A, Oliden VC, Liu Q, Shah S, Bhagia P, Kato K. Pembrolizumab plus chemotherapy versus chemotherapy alone for first-line treatment of advanced oesophageal cancer (KEYNOTE-590): a randomised, placebo-controlled, phase 3 study. Lancet. 2021;398(10302):759–71. 34454674 10.1016/S0140-6736(21)01234-4

[13] Wang ZX, Cui C, Yao J, Zhang Y, Li M, Feng J, Yang S, Fan Y, Shi J, Zhang X, Shen L, Shu Y, Wang C, Dai T, Mao T, Chen L, Guo Z, Liu B, Pan H, Cang S, Jiang Y, Wang J, Ye M, Chen Z, Jiang D, Lin Q, Ren W, Wang J, Wu L, Xu Y, Miao Z, Sun M, Xie C, Liu Y, Wang Q, Zhao L, Li Q, Huang C, Jiang K, Yang K, Li D, Liu Y, Zhu Z, Chen R, Jia L, Li W, Liao W, Liu HX, Ma D, Ma J, Qin Y, Shi Z, Wei Q, Xiao K, Zhang Y, Zhang Y, Chen X, Dai G, He J, Li J, Li G, Liu Y, Liu Z, Yuan X, Zhang J, Fu Z, He Y, Ju F, Liu Z, Tang P, Wang T, Wang W, Zhang J, Luo X, Tang X, May R, Feng H, Yao S, Keegan P, Xu RH, Wang F. Toripalimab plus chemotherapy in treatment-naive, advanced esophageal squamous cell carcinoma (JUPITER-06): A multi-center phase 3 trial. Cancer Cell. 2022;40(3):277–88. e3. 35245446 10.1016/j.ccell.2022.02.007

[14] Hoy SM, Sintilimab. First Global Approval Drugs. 2019;79(3):341–6. 30742278 10.1007/s40265-019-1066-z

[15] Markham A, Keam SJ, Camrelizumab. First Global Approval Drugs. 2019;79(12):1355–61. 31313098 10.1007/s40265-019-01167-0

[16] Keam SJ, Toripalimab. First Global Approval. Drugs. 2019;79(5):573–8. Epub 2019/02/26. 30805896 10.1007/s40265-019-01076-2

[17] Lu Z, Wang J, Shu Y, Liu L, Kong L, Yang L, Wang B, Sun G, Ji Y, Cao G, Liu H, Cui T, Li N, Qiu W, Li G, Hou X, Luo H, Xue L, Zhang Y, Yue W, Liu Z, Wang X, Gao S, Pan Y, Galais MP, Zaanan A, Ma Z, Li H, Wang Y, Shen L. group O-s. Sintilimab versus placebo in combination with chemotherapy as first line treatment for locally advanced or metastatic oesophageal squamous cell carcinoma (ORIENT-15): multicentre, randomised, double blind, phase 3 trial. BMJ (Clinical Res ed). 2022;377:e068714. 10.1136/bmj-2021-068714. Epub 2022/04/21. 10.1136/bmj-2021-068714 PMC9016493 35440464

[18] Al-Batran SE, Van Cutsem E, Oh SC, Bodoky G, Shimada Y, Hironaka S, Sugimoto N, Lipatov ON, Kim TY, Cunningham D, Rougier P, Muro K, Liepa AM, Chandrawansa K, Emig M, Ohtsu A, Wilke H. Quality-of-life and performance status results from the phase III RAINBOW study of ramucirumab plus paclitaxel versus placebo plus paclitaxel in patients with previously treated gastric or gastroesophageal junction adenocarcinoma. Annals oncology: official J Eur Soc Med Oncol. 2016;27(4):673–9. 10.1093/annonc/mdv625 PMC4803452 26747859

[19] Hoyle M, Green C. Cost-Effectiveness of Temsirolimus for First Line Treatment of Advanced Renal Cell. Value Health. 2010;13(1):61–8. 19804430 10.1111/j.1524-4733.2009.00617.x

[20] Liu S, Dou L, Wang K, Shi Z, Wang R, Zhu X, Song Z, Li S. Cost-effectiveness analysis of nivolumab combination therapy in the first-line treatment for advanced esophageal squamous-cell carcinoma. Front Oncol. 2022;12:899966. 35936686 10.3389/fonc.2022.899966 PMC9353037

[21] Xie FZP. Cost–effectiveness analysis of nivolumab in the second-line treatment for advanced esophageal squamous cell carcinoma. Future Oncol. 2020;16(17):1189–98. 32407173 10.2217/fon-2019-0821

[22] Lin YT, Liu TX, Chen J, Wang C, Chen Y. Cost-Effectiveness of Nivolumab Immunotherapy vs. Paclitaxel or Docetaxel Chemotherapy as Second-Line Therapy in Advanced Esophageal Squamous Cell Carcinoma in China. Front public health. 2022;10:923619. 35844891 10.3389/fpubh.2022.923619 PMC9277084

[23] Zhan M, Xu T, Zheng H, He Z. Cost-Effectiveness Analysis of Pembrolizumab in Patients With Advanced Esophageal Cancer Based on the KEYNOTE-181 Study. Front public health. 2022;10:790225. 35309225 10.3389/fpubh.2022.790225 PMC8924414

[24] Zheng Z, Lin J, Zhu H, Cai H. Cost-Effectiveness Analysis of Pembrolizumab Plus Chemotherapy vs. Chemotherapy Alone as First-Line Treatment in Patients With Esophageal Squamous Cell Carcinoma and PD-L1 CPS of 10 or More. Front public health. 2022;10:893387. 35774581 10.3389/fpubh.2022.893387 PMC9237361

[25] Zhu Y, Liu K, Ding D, Zhou Y, Peng L. Pembrolizumab Plus Chemotherapy as First-Line Treatment for Advanced Esophageal Cancer: A Cost-Effectiveness Analysis. Adv therapy. 2022;39(6):2614–29. 10.1007/s12325-022-02101-9 35394255

[26] Zeng F-y, Ye Z-m, Wang H-l, Xu Z, Zhou Q, Li H. A cost-effectiveness analysis of toripalimab plus TP versus TP for the first-line treatment of advanced esophageal cancer. 2022.

[27] Zhang Q, Wu P, He X, Ding Y, Shu Y. Cost-Effectiveness Analysis of Camrelizumab vs. Placebo Added to Chemotherapy as First-Line Therapy for Advanced or Metastatic Esophageal Squamous Cell Carcinoma in China. Front Oncol. 2021;11:790373. 34926306 10.3389/fonc.2021.790373 PMC8671697

[28] Lin YT, Chen Y, Liu TX, Kuang F, Huang P. Cost-Effectiveness Analysis of Camrelizumab Immunotherapy versus Docetaxel or Irinotecan Chemotherapy as Second-Line Therapy for Advanced or Metastatic Esophageal Squamous Cell Carcinoma. Cancer Manag Res. 2021;13:8219–30. 34754242 10.2147/CMAR.S335515 PMC8572144

[29] Cai H, Xu B, Li N, Zheng B, Zheng Z, Liu M. Cost-Effectiveness Analysis of Camrelizumab Versus Chemotherapy as Second-Line Treatment of Advanced or Metastatic Esophageal Squamous Cell Carcinoma. Front Pharmacol. 2021;12:732912. 34867339 10.3389/fphar.2021.732912 PMC8634950

[30] Li L, Liu X, Huang J, Liu Y, Huang L, Feng Y. Cost-effectiveness of camrelizumab versus chemotherapy for the treatment of advanced or metastatic esophageal squamous cell carcinoma. J Gastrointest Oncol. 2022;13(1):40–8. 35284115 10.21037/jgo-21-870 PMC8899749

